# An amide cyclo­phane

**DOI:** 10.1107/S1600536814015621

**Published:** 2014-07-11

**Authors:** Vijayan Viswanathan, Ayyavu Thirunarayanan, Perumal Rajakumar, Devadasan Velmurugan

**Affiliations:** a Centre of Advanced Study in Crystallography and Biophysics, University of Madras, Guindy Campus, Chennai 600 025, India; b Department of Organic Chemistry, University of Madras, Guindy Campus, Chennai 600 025, India

**Keywords:** crystal structure

## Abstract

The title compound, 8,18-dithia-2,6-diaza-13(1,4)-piperidina-1(1,2),4(1,3),7(1,2)-tribenzenaoctadecaphane-10,15-diyne-3,6-dione, C_32_H_30_N_4_O_2_S_2_, is composed of a relatively planar bis­(2-mercaptophen­yl)isophthalamide unit linked to a bridging 1,4-di(but-2-yn-1-yl)piperazine unit, forming a macrocycle. The isophthalamide ring is inclined to the outer mercaptophenyl rings by 8.18 (11) and 5.59 (10)°, while these two rings are inclined to one another by 9.10 (12)°. The piperazine ring adopts a chair conformation. There are two intra­molecular N—H⋯S hydrogen bonds generating *S*(5) ring motifs. In the crystal, mol­ecules are linked *via* C—H⋯S and C—H⋯O hydrogen bonds, forming slabs lying parallel to (001). An O atom in the isophthalamide group is disordered over two positions with an occupancy ratio of 0.41 (6):0.59 (6).

## Related literature   

For the biological activity of piperazine derivatives, see: Fun *et al.* (2011 ▶); Kavitha *et al.* (2013 ▶). For standard bond lengths, see: Allen *et al.* (1987 ▶).
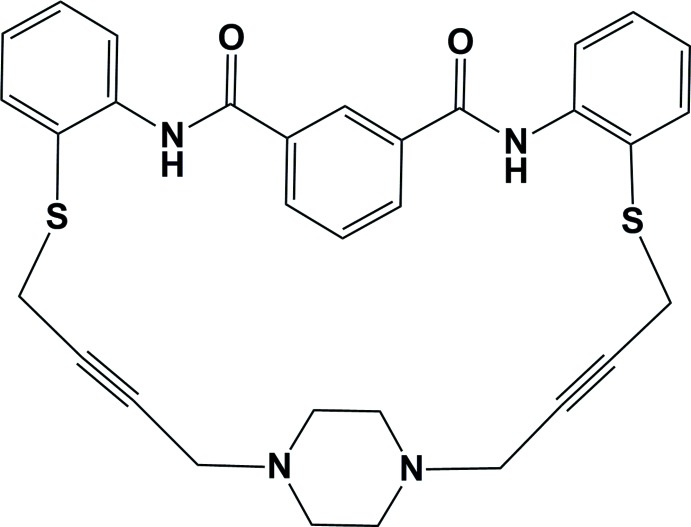


## Experimental   

### 

#### Crystal data  

C_32_H_30_N_4_O_2_S_2_
*M* *_r_* = 566.72
Monoclinic, 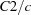

*a* = 23.0760 (5) Å
*b* = 9.9380 (3) Å
*c* = 27.0341 (7) Å
β = 108.428 (2)°
*V* = 5881.8 (3) Å^3^
*Z* = 8
Mo *K*α radiation
μ = 0.22 mm^−1^
*T* = 293 K
0.30 × 0.25 × 0.20 mm

#### Data collection  

Bruker SMART APEXII area-detector diffractometer
Absorption correction: multi-scan (*SADABS*; Bruker, 2008 ▶) *T*_min_ = 0.380, *T*_max_ = 0.745
27488 measured reflections
7294 independent reflections
4040 reflections with *I* > 2σ(*I*)
*R*_int_ = 0.036

#### Refinement  

*R*[*F* ^2^ > 2σ(*F* ^2^)] = 0.042
*wR*(*F* ^2^) = 0.117
*S* = 1.01
7294 reflections
377 parameters
2 restraints
H atoms treated by a mixture of independent and constrained refinement
Δρ_max_ = 0.20 e Å^−3^
Δρ_min_ = −0.23 e Å^−3^

### 

Data collection: *APEX2* (Bruker, 2008 ▶); cell refinement: *SAINT* (Bruker, 2008 ▶); data reduction: *SAINT*; program(s) used to solve structure: *SHELXS97* (Sheldrick, 2008 ▶); program(s) used to refine structure: *SHELXL97* (Sheldrick, 2008 ▶); molecular graphics: *ORTEP-3 for Windows* (Farrugia, 2012 ▶); software used to prepare material for publication: *SHELXL97* and *PLATON* (Spek, 2009 ▶).

## Supplementary Material

Crystal structure: contains datablock(s) global, I. DOI: 10.1107/S1600536814015621/su2737sup1.cif

Structure factors: contains datablock(s) I. DOI: 10.1107/S1600536814015621/su2737Isup2.hkl

CCDC reference: 1012131

Additional supporting information:  crystallographic information; 3D view; checkCIF report

## Figures and Tables

**Table 1:** Hydrogen-bond geometry (Å, °)

*D*—H⋯*A*	*D*—H	H⋯*A*	*D*⋯*A*	*D*—H⋯*A*
N2—H2*A*⋯S1	0.83 (2)	2.46 (2)	2.9621 (18)	120 (2)
N3—H3*A*⋯S2	0.83 (2)	2.49 (2)	2.9905 (19)	120 (2)
C21—H21*B*⋯O2*A* ^i^	0.97	2.57	3.43 (3)	148
C21—H21*B*⋯O2*B* ^i^	0.97	2.34	3.230 (11)	152
C24—H24*A*⋯O1^ii^	0.97	2.54	3.505 (3)	171
C26—H26*B*⋯S2^ii^	0.97	2.85	3.632 (2)	139
C32—H32*B*⋯O1^iii^	0.97	2.49	3.146 (3)	125

Symmetry codes: (i) 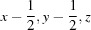; (ii) 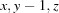; (iii) 
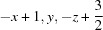
.

**Fig. 1.**
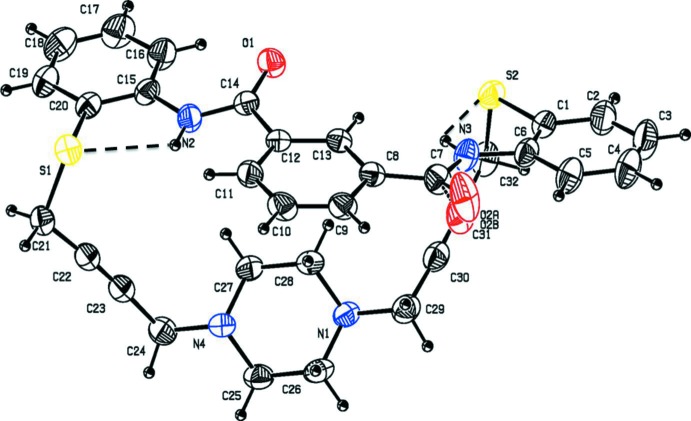
The molecular structure of the title molecule, with atom labelling. Displacement ellipsoids are drawn at the 50% probability level. The intramolecular N-H···S hydrogen bonds are shown as dashed lines (see Table 1 for details).

**Fig. 2.**
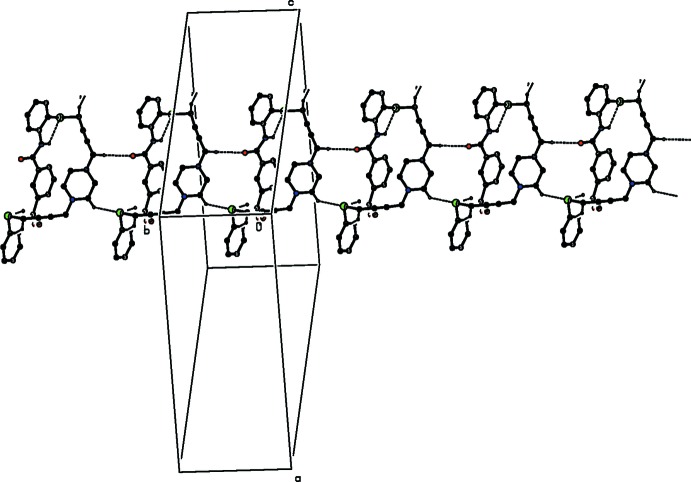
A partial view of the crystal packing of the title compound. Hydrogen bonds are shown as dashed lines (see Table 1 for details; H-atoms not involved in hydrogen bonding have been omitted for clarity).

## supplementary crystallographic information

### S1. Comment

Piperazine derivatives are popular in organic synthesis and for their applications in biology and medicine. They are known to exhibit antibacterial and antimicrobial activities (Fun *et al.*, 2011). They are also amongst the most important building blocks found in biologically active compounds in a number of different therapeutic areas and a review about the current pharmacological and toxicological information for piperazine derivatives is available (Kavitha *et al.*, 2013).

The molecular structure of the title molecule is shown in Fig. 1. It is composed of a relatively planar bis(2-mercaptophenyl)isophthalamide moiety linked to a bridging 1,4-di(but-2-yn-1-yl)piperazine unit forming a macrocycle. The isophthalamide ring (C8-C13) is inclined to the outer mercaptophenyl rings (C1-C6 and C15-C20) by 8.18 (11) and 5.59 (10) °, respectively. The two mercaptophenyl rings (C1-C6 and C15-C20) are inclined to one another by 9.10 (12) °. The S2–C1 and S1–C20 bond lengths are 1.773 (2) Å and 1.777 (2) Å, respectively, similar to the standard value of 1.769 (9) Å (Allen *et al.*, 1987). There are two intramolecular N—H···S hydrogen bonds generating S(5) ring motifs (Fig. 1 and Table 1).

The but-2-yne group is linear and connects the piperazine group to the bis(2-mercaptophenyl)isophthalamide moiety. The piperazine ring (N1/N4/C25-C28) adopts a chair conformation.

In the crystal, molecules are linked via C—H···S and C—H···O hydrogen bonds forming slabs lying parallel to (001); see Table 1 and Fig. 2.

### S2. Experimental

A mixture of precyclophane diyne (0.2 g, 3.98 mmol), piperazine (0.04 g, 3.98 mmol), formaldehyde (0.02 g, 7.96 mmol) from 37-41% formalin solution and CuCl (0.04 g, 3.98 mmol) in dioxane (30 mL) was refluxed at 363 K for 2 h under a nitrogen atmosphere by the Multi Components Reaction (MCR) technique. After the reaction was complete, the solvent was removed under reduced pressure and the residue was extracted with CHCl_3_ (3 × 100 mL), washed with water (2 × 100 mL), brine (150 mL) and dried over anhydrous Na_2_SO_4_. The solvent was removed and the crude product was purified by column chromatography on silica gel using CHCl_3_/MeOH (24:1) as eluent. After purification the piperazinophane was recrystallised in MeOH by slow evaporation yielding block-like colourless crystals.

### S3. Refinement

N-bound H atoms were refined with distance restraints: N-H = 0.86 (2) Å with U_iso_(H) = 1.2U_eq_(N). The C-bound H atoms were positioned geometrically (C–H = 0.93–0.97 Å) and allowed to ride on their parent atoms, with U_iso_(H) = 1.5U_eq_(C-methyl) and = 1.2U_eq_(C) for other H atoms. In the isophthalamide group atom O2 is disordered over two positions with an occupancy ratio of 0.41 (6):0.59 (6).

### Crystal data

**Table:**

C_32_H_30_N_4_O_2_S_2_	*F*(000) = 2384
*M**_r_* = 566.72	*D*_x_ = 1.280 Mg m^−^^3^
Monoclinic, *C*2/*c*	Mo *K*α radiation, λ = 0.71073 Å
Hall symbol: -C 2yc	Cell parameters from 7294 reflections
*a* = 23.0760 (5) Å	θ = 1.6–28.3°
*b* = 9.9380 (3) Å	µ = 0.22 mm^−^^1^
*c* = 27.0341 (7) Å	*T* = 293 K
β = 108.428 (2)°	Block, colourless
*V* = 5881.8 (3) Å^3^	0.30 × 0.25 × 0.20 mm
*Z* = 8	

### Data collection

**Table:**

Bruker SMART APEXII area-detector diffractometer	7294 independent reflections
Radiation source: fine-focus sealed tube	4040 reflections with *I* > 2σ(*I*)
Graphite monochromator	*R*_int_ = 0.036
ω and φ scans	θ_max_ = 28.3°, θ_min_ = 1.6°
Absorption correction: multi-scan (*SADABS*; Bruker, 2008)	*h* = −30→30
*T*_min_ = 0.380, *T*_max_ = 0.745	*k* = −13→13
27488 measured reflections	*l* = −34→35

### Refinement

**Table:**

Refinement on *F*^2^	Primary atom site location: structure-invariant direct methods
Least-squares matrix: full	Secondary atom site location: difference Fourier map
*R*[*F*^2^ > 2σ(*F*^2^)] = 0.042	Hydrogen site location: inferred from neighbouring sites
*wR*(*F*^2^) = 0.117	H atoms treated by a mixture of independent and constrained refinement
*S* = 1.01	*w* = 1/[σ^2^(*F*_o_^2^) + (0.0438*P*)^2^ + 2.0132*P*] where *P* = (*F*_o_^2^ + 2*F*_c_^2^)/3
7294 reflections	(Δ/σ)_max_ = 0.001
377 parameters	Δρ_max_ = 0.20 e Å^−^^3^
2 restraints	Δρ_min_ = −0.23 e Å^−^^3^

### Special details

**Table:**

Geometry. All esds (except the esd in the dihedral angle between two l.s. planes) are estimated using the full covariance matrix. The cell esds are taken into account individually in the estimation of esds in distances, angles and torsion angles; correlations between esds in cell parameters are only used when they are defined by crystal symmetry. An approximate (isotropic) treatment of cell esds is used for estimating esds involving l.s. planes.
Refinement. Refinement of F^2^ against ALL reflections. The weighted R-factor wR and goodness of fit S are based on F^2^, conventional R-factors R are based on F, with F set to zero for negative F^2^. The threshold expression of F^2^ > 2sigma(F^2^) is used only for calculating R-factors(gt) etc. and is not relevant to the choice of reflections for refinement. R-factors based on F^2^ are statistically about twice as large as those based on F, and R- factors based on ALL data will be even larger.

### Fractional atomic coordinates and isotropic or equivalent isotropic displacement parameters (Å^2^)

**Table:**

	*x*	*y*	*z*	*U*_iso_*/*U*_eq_	Occ. (<1)
S1	0.41059 (2)	0.21672 (6)	1.01916 (2)	0.06329 (16)	
S2	0.58670 (2)	0.64936 (6)	0.76454 (2)	0.06810 (17)	
O1	0.47117 (6)	0.54558 (14)	0.89655 (5)	0.0671 (4)	
O2A	0.7513 (7)	0.440 (3)	0.9253 (10)	0.108 (5)	0.41 (6)
O2B	0.7544 (4)	0.482 (3)	0.9314 (5)	0.115 (4)	0.59 (6)
N1	0.57053 (7)	0.08419 (16)	0.81521 (6)	0.0584 (4)	
N2	0.45152 (7)	0.37111 (18)	0.94254 (7)	0.0634 (5)	
H2A	0.4674 (9)	0.3112 (19)	0.9642 (8)	0.076*	
N3	0.67548 (7)	0.5408 (2)	0.86248 (7)	0.0650 (5)	
H3A	0.6377 (7)	0.544 (2)	0.8508 (8)	0.078*	
N4	0.50216 (7)	−0.03836 (15)	0.87441 (6)	0.0541 (4)	
C1	0.66745 (9)	0.6485 (2)	0.78117 (9)	0.0625 (5)	
C2	0.69417 (11)	0.7074 (3)	0.74746 (11)	0.0866 (7)	
H2	0.6695	0.7458	0.7166	0.104*	
C3	0.75672 (13)	0.7100 (3)	0.75881 (13)	0.1039 (9)	
H3	0.7744	0.7507	0.7361	0.125*	
C4	0.79235 (11)	0.6519 (3)	0.80401 (13)	0.0995 (9)	
H4	0.8346	0.6519	0.8115	0.119*	
C5	0.76738 (10)	0.5931 (3)	0.83875 (10)	0.0825 (7)	
H5	0.7925	0.5540	0.8693	0.099*	
C6	0.70420 (8)	0.5926 (2)	0.82788 (9)	0.0618 (5)	
C7	0.69931 (9)	0.4820 (2)	0.90941 (9)	0.0660 (6)	
C8	0.65534 (8)	0.43753 (18)	0.93660 (8)	0.0536 (5)	
C9	0.67931 (9)	0.3618 (2)	0.98150 (9)	0.0657 (6)	
H9	0.7211	0.3448	0.9937	0.079*	
C10	0.64258 (10)	0.3116 (2)	1.00816 (9)	0.0709 (6)	
H10	0.6594	0.2596	1.0379	0.085*	
C11	0.58065 (9)	0.3380 (2)	0.99107 (8)	0.0622 (5)	
H11	0.5558	0.3034	1.0092	0.075*	
C12	0.55554 (8)	0.41599 (17)	0.94693 (7)	0.0484 (4)	
C13	0.59316 (8)	0.46595 (18)	0.92026 (7)	0.0497 (4)	
H13	0.5766	0.5194	0.8909	0.060*	
C14	0.48890 (8)	0.45151 (19)	0.92630 (7)	0.0517 (4)	
C15	0.38767 (8)	0.3776 (2)	0.93183 (8)	0.0578 (5)	
C16	0.34978 (10)	0.4520 (2)	0.89092 (9)	0.0770 (7)	
H16	0.3663	0.5021	0.8696	0.092*	
C17	0.28782 (11)	0.4512 (3)	0.88210 (11)	0.0917 (8)	
H17	0.2625	0.5006	0.8545	0.110*	
C18	0.26290 (11)	0.3792 (3)	0.91326 (12)	0.0942 (8)	
H18	0.2209	0.3793	0.9068	0.113*	
C19	0.30033 (10)	0.3061 (2)	0.95441 (10)	0.0763 (6)	
H19	0.2834	0.2577	0.9759	0.092*	
C20	0.36274 (8)	0.3043 (2)	0.96403 (8)	0.0575 (5)	
C21	0.39422 (9)	0.0419 (2)	1.00030 (8)	0.0623 (5)	
H21A	0.4140	−0.0144	1.0302	0.075*	
H21B	0.3505	0.0277	0.9915	0.075*	
C22	0.41344 (9)	−0.0032 (2)	0.95642 (9)	0.0573 (5)	
C23	0.42885 (9)	−0.0462 (2)	0.92174 (9)	0.0590 (5)	
C24	0.44899 (10)	−0.1063 (2)	0.88040 (8)	0.0651 (5)	
H24A	0.4587	−0.2003	0.8884	0.078*	
H24B	0.4158	−0.1020	0.8477	0.078*	
C25	0.53008 (10)	−0.1175 (2)	0.84244 (9)	0.0685 (6)	
H25A	0.5012	−0.1287	0.8077	0.082*	
H25B	0.5408	−0.2061	0.8577	0.082*	
C26	0.58637 (10)	−0.0470 (2)	0.83922 (9)	0.0695 (6)	
H26A	0.6153	−0.0366	0.8739	0.083*	
H26B	0.6056	−0.1007	0.8188	0.083*	
C27	0.48647 (9)	0.0944 (2)	0.85116 (9)	0.0638 (5)	
H27A	0.4686	0.1480	0.8726	0.077*	
H27B	0.4564	0.0854	0.8169	0.077*	
C28	0.54223 (9)	0.1640 (2)	0.84643 (9)	0.0648 (6)	
H28A	0.5309	0.2514	0.8303	0.078*	
H28B	0.5713	0.1779	0.8809	0.078*	
C29	0.62297 (9)	0.1516 (2)	0.80817 (9)	0.0725 (6)	
H29A	0.6431	0.0916	0.7905	0.087*	
H29B	0.6518	0.1733	0.8420	0.087*	
C30	0.60525 (10)	0.2749 (3)	0.77769 (9)	0.0668 (6)	
C31	0.58869 (9)	0.3744 (3)	0.75342 (9)	0.0651 (6)	
C32	0.56795 (10)	0.4989 (2)	0.72467 (8)	0.0748 (6)	
H32A	0.5240	0.4946	0.7086	0.090*	
H32B	0.5861	0.5057	0.6969	0.090*	

### Atomic displacement parameters (Å^2^)

**Table:**

	*U*^11^	*U*^22^	*U*^33^	*U*^12^	*U*^13^	*U*^23^
S1	0.0621 (3)	0.0687 (3)	0.0562 (3)	−0.0058 (3)	0.0146 (2)	0.0010 (3)
S2	0.0538 (3)	0.0752 (4)	0.0786 (4)	0.0148 (3)	0.0256 (3)	0.0111 (3)
O1	0.0593 (8)	0.0693 (9)	0.0674 (9)	0.0087 (7)	0.0123 (7)	0.0201 (8)
O2A	0.035 (5)	0.131 (11)	0.160 (10)	0.023 (6)	0.032 (6)	0.061 (7)
O2B	0.050 (3)	0.167 (9)	0.104 (5)	−0.038 (5)	−0.010 (3)	0.051 (5)
N1	0.0518 (9)	0.0574 (10)	0.0665 (11)	0.0045 (8)	0.0195 (8)	−0.0074 (8)
N2	0.0491 (9)	0.0689 (12)	0.0744 (12)	0.0115 (8)	0.0229 (9)	0.0232 (9)
N3	0.0378 (8)	0.0918 (13)	0.0638 (11)	−0.0020 (9)	0.0140 (8)	0.0025 (10)
N4	0.0568 (9)	0.0477 (9)	0.0555 (10)	0.0057 (7)	0.0146 (8)	−0.0030 (8)
C1	0.0536 (11)	0.0650 (13)	0.0762 (15)	0.0019 (10)	0.0308 (11)	−0.0003 (11)
C2	0.0780 (16)	0.1015 (19)	0.0924 (18)	0.0038 (14)	0.0444 (14)	0.0149 (15)
C3	0.0777 (18)	0.134 (3)	0.118 (2)	−0.0075 (17)	0.0565 (18)	0.016 (2)
C4	0.0535 (14)	0.137 (3)	0.119 (2)	−0.0141 (15)	0.0430 (16)	−0.006 (2)
C5	0.0482 (12)	0.114 (2)	0.0867 (17)	−0.0087 (13)	0.0239 (12)	−0.0074 (15)
C6	0.0466 (11)	0.0719 (13)	0.0715 (14)	−0.0064 (10)	0.0250 (10)	−0.0109 (11)
C7	0.0454 (11)	0.0712 (14)	0.0750 (15)	−0.0064 (11)	0.0098 (11)	0.0034 (12)
C8	0.0461 (10)	0.0476 (10)	0.0620 (12)	−0.0014 (8)	0.0101 (9)	−0.0022 (9)
C9	0.0483 (11)	0.0615 (13)	0.0798 (15)	0.0079 (10)	0.0094 (10)	0.0108 (11)
C10	0.0644 (13)	0.0672 (14)	0.0718 (15)	0.0120 (11)	0.0082 (11)	0.0211 (11)
C11	0.0606 (12)	0.0617 (12)	0.0640 (13)	0.0072 (10)	0.0194 (10)	0.0130 (10)
C12	0.0501 (10)	0.0419 (10)	0.0515 (11)	0.0050 (8)	0.0138 (9)	−0.0006 (8)
C13	0.0491 (10)	0.0452 (10)	0.0500 (11)	−0.0003 (8)	0.0087 (8)	−0.0014 (8)
C14	0.0541 (11)	0.0515 (11)	0.0490 (11)	0.0065 (9)	0.0156 (9)	−0.0003 (9)
C15	0.0459 (10)	0.0674 (13)	0.0605 (12)	0.0093 (9)	0.0176 (9)	0.0024 (10)
C16	0.0611 (13)	0.0964 (17)	0.0723 (15)	0.0196 (12)	0.0192 (11)	0.0208 (13)
C17	0.0590 (14)	0.116 (2)	0.0890 (18)	0.0270 (14)	0.0080 (13)	0.0195 (17)
C18	0.0475 (13)	0.118 (2)	0.112 (2)	0.0165 (14)	0.0184 (14)	0.0008 (18)
C19	0.0549 (13)	0.0916 (17)	0.0885 (17)	0.0025 (12)	0.0313 (12)	−0.0010 (14)
C20	0.0491 (10)	0.0642 (12)	0.0596 (12)	0.0033 (9)	0.0176 (9)	−0.0051 (10)
C21	0.0569 (12)	0.0657 (13)	0.0643 (13)	−0.0067 (10)	0.0192 (10)	0.0061 (11)
C22	0.0478 (11)	0.0565 (12)	0.0624 (13)	−0.0020 (9)	0.0101 (10)	0.0099 (10)
C23	0.0534 (11)	0.0564 (12)	0.0627 (14)	−0.0017 (9)	0.0117 (10)	0.0056 (11)
C24	0.0688 (13)	0.0605 (13)	0.0627 (13)	−0.0048 (11)	0.0159 (11)	−0.0036 (11)
C25	0.0812 (15)	0.0525 (12)	0.0730 (15)	0.0038 (11)	0.0259 (12)	−0.0107 (11)
C26	0.0676 (13)	0.0595 (13)	0.0838 (16)	0.0143 (11)	0.0274 (12)	−0.0113 (12)
C27	0.0598 (12)	0.0596 (12)	0.0746 (14)	0.0140 (10)	0.0249 (11)	0.0068 (11)
C28	0.0679 (13)	0.0508 (11)	0.0794 (15)	0.0084 (10)	0.0287 (11)	−0.0017 (11)
C29	0.0562 (12)	0.0752 (15)	0.0877 (17)	0.0028 (11)	0.0252 (12)	−0.0067 (13)
C30	0.0542 (12)	0.0773 (16)	0.0728 (15)	−0.0072 (12)	0.0257 (11)	−0.0155 (13)
C31	0.0503 (12)	0.0834 (17)	0.0638 (14)	−0.0069 (12)	0.0213 (10)	−0.0138 (13)
C32	0.0618 (13)	0.1047 (18)	0.0559 (13)	−0.0025 (12)	0.0157 (11)	0.0029 (13)

### Geometric parameters (Å, º)

**Table:**

S1—C20	1.777 (2)	C12—C13	1.384 (3)
S1—C21	1.817 (2)	C12—C14	1.503 (2)
S2—C1	1.773 (2)	C13—H13	0.9300
S2—C32	1.814 (2)	C15—C16	1.387 (3)
O1—C14	1.217 (2)	C15—C20	1.391 (3)
O2A—C7	1.215 (11)	C16—C17	1.373 (3)
O2B—C7	1.222 (9)	C16—H16	0.9300
N1—C29	1.448 (3)	C17—C18	1.363 (4)
N1—C26	1.451 (3)	C17—H17	0.9300
N1—C28	1.454 (2)	C18—C19	1.380 (3)
N2—C14	1.347 (2)	C18—H18	0.9300
N2—C15	1.411 (2)	C19—C20	1.380 (3)
N2—H2A	0.833 (15)	C19—H19	0.9300
N3—C7	1.346 (3)	C21—C22	1.462 (3)
N3—C6	1.404 (3)	C21—H21A	0.9700
N3—H3A	0.828 (15)	C21—H21B	0.9700
N4—C24	1.454 (3)	C22—C23	1.183 (3)
N4—C27	1.457 (2)	C23—C24	1.466 (3)
N4—C25	1.461 (2)	C24—H24A	0.9700
C1—C2	1.382 (3)	C24—H24B	0.9700
C1—C6	1.395 (3)	C25—C26	1.503 (3)
C2—C3	1.378 (3)	C25—H25A	0.9700
C2—H2	0.9300	C25—H25B	0.9700
C3—C4	1.367 (4)	C26—H26A	0.9700
C3—H3	0.9300	C26—H26B	0.9700
C4—C5	1.378 (4)	C27—C28	1.502 (3)
C4—H4	0.9300	C27—H27A	0.9700
C5—C6	1.393 (3)	C27—H27B	0.9700
C5—H5	0.9300	C28—H28A	0.9700
C7—C8	1.496 (3)	C28—H28B	0.9700
C8—C9	1.386 (3)	C29—C30	1.461 (3)
C8—C13	1.390 (2)	C29—H29A	0.9700
C9—C10	1.369 (3)	C29—H29B	0.9700
C9—H9	0.9300	C30—C31	1.181 (3)
C10—C11	1.381 (3)	C31—C32	1.459 (3)
C10—H10	0.9300	C32—H32A	0.9700
C11—C12	1.387 (3)	C32—H32B	0.9700
C11—H11	0.9300		
			
C20—S1—C21	102.37 (10)	C18—C17—C16	121.0 (2)
C1—S2—C32	100.43 (10)	C18—C17—H17	119.5
C29—N1—C26	111.83 (16)	C16—C17—H17	119.5
C29—N1—C28	111.80 (16)	C17—C18—C19	119.7 (2)
C26—N1—C28	109.05 (16)	C17—C18—H18	120.1
C14—N2—C15	129.86 (17)	C19—C18—H18	120.1
C14—N2—H2A	117.9 (15)	C18—C19—C20	120.6 (2)
C15—N2—H2A	112.1 (15)	C18—C19—H19	119.7
C7—N3—C6	130.45 (17)	C20—C19—H19	119.7
C7—N3—H3A	116.3 (16)	C19—C20—C15	119.24 (19)
C6—N3—H3A	113.1 (16)	C19—C20—S1	119.93 (17)
C24—N4—C27	111.41 (15)	C15—C20—S1	120.74 (14)
C24—N4—C25	111.20 (15)	C22—C21—S1	115.54 (14)
C27—N4—C25	109.16 (16)	C22—C21—H21A	108.4
C2—C1—C6	119.7 (2)	S1—C21—H21A	108.4
C2—C1—S2	118.89 (18)	C22—C21—H21B	108.4
C6—C1—S2	121.39 (15)	S1—C21—H21B	108.4
C3—C2—C1	121.1 (3)	H21A—C21—H21B	107.5
C3—C2—H2	119.5	C23—C22—C21	176.6 (2)
C1—C2—H2	119.5	C22—C23—C24	176.9 (2)
C4—C3—C2	118.9 (2)	N4—C24—C23	112.22 (16)
C4—C3—H3	120.6	N4—C24—H24A	109.2
C2—C3—H3	120.6	C23—C24—H24A	109.2
C3—C4—C5	121.7 (2)	N4—C24—H24B	109.2
C3—C4—H4	119.1	C23—C24—H24B	109.2
C5—C4—H4	119.1	H24A—C24—H24B	107.9
C4—C5—C6	119.5 (2)	N4—C25—C26	109.52 (16)
C4—C5—H5	120.2	N4—C25—H25A	109.8
C6—C5—H5	120.2	C26—C25—H25A	109.8
C5—C6—C1	119.1 (2)	N4—C25—H25B	109.8
C5—C6—N3	122.9 (2)	C26—C25—H25B	109.8
C1—C6—N3	117.99 (17)	H25A—C25—H25B	108.2
O2A—C7—N3	123.2 (9)	N1—C26—C25	110.17 (17)
O2B—C7—N3	120.8 (7)	N1—C26—H26A	109.6
O2A—C7—C8	118.2 (9)	C25—C26—H26A	109.6
O2B—C7—C8	121.5 (6)	N1—C26—H26B	109.6
N3—C7—C8	116.93 (17)	C25—C26—H26B	109.6
C9—C8—C13	118.33 (18)	H26A—C26—H26B	108.1
C9—C8—C7	116.48 (17)	N4—C27—C28	110.57 (16)
C13—C8—C7	125.19 (18)	N4—C27—H27A	109.5
C10—C9—C8	121.13 (19)	C28—C27—H27A	109.5
C10—C9—H9	119.4	N4—C27—H27B	109.5
C8—C9—H9	119.4	C28—C27—H27B	109.5
C9—C10—C11	120.15 (19)	H27A—C27—H27B	108.1
C9—C10—H10	119.9	N1—C28—C27	110.61 (17)
C11—C10—H10	119.9	N1—C28—H28A	109.5
C10—C11—C12	120.04 (19)	C27—C28—H28A	109.5
C10—C11—H11	120.0	N1—C28—H28B	109.5
C12—C11—H11	120.0	C27—C28—H28B	109.5
C13—C12—C11	119.24 (17)	H28A—C28—H28B	108.1
C13—C12—C14	117.33 (16)	N1—C29—C30	111.46 (17)
C11—C12—C14	123.43 (17)	N1—C29—H29A	109.3
C12—C13—C8	121.06 (17)	C30—C29—H29A	109.3
C12—C13—H13	119.5	N1—C29—H29B	109.3
C8—C13—H13	119.5	C30—C29—H29B	109.3
O1—C14—N2	123.71 (18)	H29A—C29—H29B	108.0
O1—C14—C12	121.12 (18)	C31—C30—C29	177.3 (2)
N2—C14—C12	115.16 (16)	C30—C31—C32	178.5 (2)
C16—C15—C20	119.81 (18)	C31—C32—S2	113.96 (15)
C16—C15—N2	122.85 (19)	C31—C32—H32A	108.8
C20—C15—N2	117.34 (17)	S2—C32—H32A	108.8
C17—C16—C15	119.7 (2)	C31—C32—H32B	108.8
C17—C16—H16	120.2	S2—C32—H32B	108.8
C15—C16—H16	120.2	H32A—C32—H32B	107.7
			
C32—S2—C1—C2	86.2 (2)	C13—C12—C14—O1	−18.3 (3)
C32—S2—C1—C6	−95.30 (19)	C11—C12—C14—O1	160.92 (19)
C6—C1—C2—C3	1.0 (4)	C13—C12—C14—N2	160.74 (17)
S2—C1—C2—C3	179.6 (2)	C11—C12—C14—N2	−20.0 (3)
C1—C2—C3—C4	0.7 (4)	C14—N2—C15—C16	17.0 (3)
C2—C3—C4—C5	−1.2 (5)	C14—N2—C15—C20	−163.6 (2)
C3—C4—C5—C6	−0.1 (4)	C20—C15—C16—C17	−0.8 (3)
C4—C5—C6—C1	1.8 (4)	N2—C15—C16—C17	178.5 (2)
C4—C5—C6—N3	−176.7 (2)	C15—C16—C17—C18	0.5 (4)
C2—C1—C6—C5	−2.2 (3)	C16—C17—C18—C19	0.2 (4)
S2—C1—C6—C5	179.22 (17)	C17—C18—C19—C20	−0.6 (4)
C2—C1—C6—N3	176.3 (2)	C18—C19—C20—C15	0.3 (3)
S2—C1—C6—N3	−2.2 (3)	C18—C19—C20—S1	176.77 (19)
C7—N3—C6—C5	−1.6 (4)	C16—C15—C20—C19	0.4 (3)
C7—N3—C6—C1	179.9 (2)	N2—C15—C20—C19	−178.96 (19)
C6—N3—C7—O2A	−15 (2)	C16—C15—C20—S1	−176.02 (17)
C6—N3—C7—O2B	9.3 (15)	N2—C15—C20—S1	4.6 (3)
C6—N3—C7—C8	180.0 (2)	C21—S1—C20—C19	72.16 (19)
O2A—C7—C8—C9	6 (2)	C21—S1—C20—C15	−111.40 (17)
O2B—C7—C8—C9	−17.5 (15)	C20—S1—C21—C22	66.21 (17)
N3—C7—C8—C9	171.90 (19)	C27—N4—C24—C23	−71.5 (2)
O2A—C7—C8—C13	−173 (2)	C25—N4—C24—C23	166.44 (17)
O2B—C7—C8—C13	162.6 (15)	C24—N4—C25—C26	−177.46 (17)
N3—C7—C8—C13	−7.9 (3)	C27—N4—C25—C26	59.2 (2)
C13—C8—C9—C10	2.5 (3)	C29—N1—C26—C25	−176.08 (18)
C7—C8—C9—C10	−177.4 (2)	C28—N1—C26—C25	59.8 (2)
C8—C9—C10—C11	−1.2 (3)	N4—C25—C26—N1	−60.9 (2)
C9—C10—C11—C12	−0.3 (3)	C24—N4—C27—C28	178.72 (17)
C10—C11—C12—C13	0.4 (3)	C25—N4—C27—C28	−58.1 (2)
C10—C11—C12—C14	−178.84 (18)	C29—N1—C28—C27	177.58 (17)
C11—C12—C13—C8	1.0 (3)	C26—N1—C28—C27	−58.2 (2)
C14—C12—C13—C8	−179.76 (16)	N4—C27—C28—N1	58.1 (2)
C9—C8—C13—C12	−2.4 (3)	C26—N1—C29—C30	172.24 (18)
C7—C8—C13—C12	177.49 (18)	C28—N1—C29—C30	−65.2 (2)
C15—N2—C14—O1	−2.6 (3)	C1—S2—C32—C31	66.79 (18)
C15—N2—C14—C12	178.37 (19)		

### Hydrogen-bond geometry (Å, º)

**Table:**

*D*—H···*A*	*D*—H	H···*A*	*D*···*A*	*D*—H···*A*
N2—H2*A*···S1	0.83 (2)	2.46 (2)	2.9621 (18)	120 (2)
N3—H3*A*···S2	0.83 (2)	2.49 (2)	2.9905 (19)	120 (2)
C21—H21*B*···O2*A*^i^	0.97	2.57	3.43 (3)	148
C21—H21*B*···O2*B*^i^	0.97	2.34	3.230 (11)	152
C24—H24*A*···O1^ii^	0.97	2.54	3.505 (3)	171
C26—H26*B*···S2^ii^	0.97	2.85	3.632 (2)	139
C32—H32*B*···O1^iii^	0.97	2.49	3.146 (3)	125

Symmetry codes: (i) *x*−1/2, *y*−1/2, *z*; (ii) *x*, *y*−1, *z*; (iii) −*x*+1, *y*, −*z*+3/2.
